# Adding Content to Contacts: Measurement of High Quality Contacts for Maternal and Newborn Health in Ethiopia, North East Nigeria, and Uttar Pradesh, India

**DOI:** 10.1371/journal.pone.0126840

**Published:** 2015-05-22

**Authors:** Tanya Marchant, Ritgak Dimka Tilley-Gyado, Tsegahun Tessema, Kultar Singh, Meenakshi Gautham, Nasir Umar, Della Berhanu, Simon Cousens, Joanna RM Armstrong Schellenberg

**Affiliations:** 1 Faculty of Infectious and Tropical Disease, London School of Hygiene & Tropical Medicine, London, United Kingdom; 2 Health Hub Ltd., Abuja, Nigeria; 3 JaRco Consulting, Addis Ababa, Ethiopia; 4 Sambodhi Research and Communications Pvt. Ltd, New Delhi, 110024, India; 5 Faculty of Epidemiology and Population Health, London School of Hygiene and Tropical Medicine, London, United Kingdom; Banaras Hindu University, INDIA

## Abstract

**Background:**

Families in high mortality settings need regular contact with high quality services, but existing population-based measurements of contacts do not reflect quality. To address this, in 2012, we designed linked household and frontline worker surveys for Gombe State, Nigeria, Ethiopia, and Uttar Pradesh, India. Using reported frequency and content of contacts, we present a method for estimating the population level coverage of high quality contacts.

**Methods and Findings:**

Linked cluster-based household and frontline health worker surveys were performed. Interviews were conducted in 40, 80 and 80 clusters in Gombe, Ethiopia, and Uttar Pradesh, respectively, including 348, 533, and 604 eligible women and 20, 76, and 55 skilled birth attendants. High quality contacts were defined as contacts during which recommended set of processes for routine health care were met. In Gombe, 61% (95% confidence interval 50-72) of women had at least one antenatal contact, 22% (14-29) delivered with a skilled birth attendant, 7% (4-9) had a post-partum check and 4% (2-8) of newborns had a post-natal check. Coverage of high quality contacts was reduced to 11% (6-16), 8% (5-11), 0%, and 0% respectively. In Ethiopia, 56% (49-63) had at least one antenatal contact, 15% (11-22) delivered with a skilled birth attendant, 3% (2-6) had a post-partum check and 4% (2-6) of newborns had a post-natal check. Coverage of high quality contacts was 4% (2-6), 4% (2-6), 0%, and 0%, respectively. In Uttar Pradesh 74% (69-79) had at least one antenatal contact, 76% (71-80) delivered with a skilled birth attendant, 54% (48-59) had a post-partum check and 19% (15-23) of newborns had a post-natal check. Coverage of high quality contacts was 6% (4-8), 4% (2-6), 0%, and 0% respectively.

**Conclusions:**

Measuring content of care to reflect the quality of contacts can reveal missed opportunities to deliver best possible health care.

## Introduction

The frequency of contacts that women and their newborns have with health care providers is increasing globally, even in some high mortality settings.[1] Increased access to the continuum of care for mothers and newborns is important for improved survival. Beyond the delivery of life-saving interventions at each stage of care, women who receive care in pregnancy are more likely to go on to have a skilled attendant at birth when the majority of deaths occur.[2–4] It has been estimated that between 13% and 33% of maternal deaths could be eliminated by the presence of a skilled attendant at delivery, as could approximately 25% of newborn deaths.[5, 6] However, increases in contact, especially delivery by a skilled attendant, has not been consistently linked to decreases in maternal or neonatal mortality in recent years,[7] a reflection in part of the low quality of care provided in many resource poor settings.[8] In the context of this increasing coverage, there is a need to focus on the quality of contacts that have the potential to save lives: antenatal care, delivery with a skilled attendant, and health checks for mother and newborn within 48 hours of birth.[9]

But integrating measures of quality in measures of contacts is challenging. Quality indicators have been proposed,[10–12] however due to measurement complexities their coverage has rarely been reported at the population level in high mortality settings. In part this is because quality of care for mothers and newborns is multi-dimensional and conceptually complex, reflecting both the provision (in terms of structure, process and outcomes) and the experience of receiving health care. One approach to simplify this complexity is to focus on the content of care received as defined by the routine processes that are recommended to occur during a contact between health care user and provider. It has been argued that these processes provide a measurable and meaningful indication of quality since they describe the potential for health gain from a given clinical scenario.[13]

In the context of maternal and newborn health care it is possible to define content of care by drawing on the global recommendations for minimum packages of routine care that should be made available to all women and infants during antenatal, intra-partum and post-natal contacts (Table 1).[14, 15] Important to note is that these refer to the behaviours that health workers carry out as part of routine care, as distinct from delivery of life-saving interventions that may occur as a result of these behaviours: for example, measurement of blood pressure during antenatal care being a process that is a necessary precursor to administration of the potentially life-saving intervention of hypertensive drug prescription to manage pre-eclampsia, if indicated.

**Table 1:** Content of care defined by routine processes for antenatal, post-partum and post-natal care, and for prevention of haemorrhage during skilled birth attendance.

Contact for maternal and newborn health	Number of criteria:	Routine processes:
Antenatal care^1^	8	Weight and height measured; blood pressure measured; urine and blood tests carried out; counselling for breastfeeding, danger signs, and birth preparedness
Prevention of haemorrhage during skilled birth attendance^2^	2	Administration of prophylactic uterotonics to prevent post-partum haemorrhage; active management of third stage of labour;
Post-partum care^3^	5	Breasts and bleeding checked; counselled on danger signs, nutrition, and family planning
Post-natal care^3^	5	Weigh newborn; check cord care; counsel caregiver on breastfeeding, thermal care and danger signs

^1^ Opportunities for Africa’s newborns: practical data, policy and programmatic support for newborn care in Africa, Section III—Chapter 2: Antenatal care. Published by WHO on behalf of The Partnership for Maternal Newborn and Child Health, 2006. Accessed at: http://www.who.int/pmnch/media/publications/aonsectionIII_2.pdf

^2^ The processes shown for skilled birth attendance reflect only the basic routine recommendations for prevention of haemorrhage amongst all women, not the full spectrum of essential care. WHO recommendations for the prevention and treatment of postpartum haemorrhage. Published by WHO, 2012, ISBN 978 92 4 154850 2. Accessed at: http://apps.who.int/iris/bitstream/10665/75411/1/9789241548502_eng.pdf?ua=1

^3^ Opportunities for Africa’s newborns: practical data, policy and programmatic support for newborn care in Africa, Section III—Chapter 4: Postnatal care. Published by WHO on behalf of The Partnership for Maternal Newborn and Child Health, 2006. Accessed at: http://www.who.int/pmnch/media/publications/aonsectionIII_4.pdf

In this article we propose a measurement method for evaluating the quality of health care for mothers and newborns that links the coverage of each type of contact to the content of care that should take place during those contacts to estimate the coverage of high quality contacts at the population level.

## Methods

### Study area

This study was performed in Gombe State of north east Nigeria, the regions of Oromia, Tigray, Amhara and Southern Nations Nationalities and Peoples (SNNP) in Ethiopia, and in six districts (Jhansi, Hardoi, CSM Nagar, Maharanjganj, Sultanpur, Raebarailly) in the state of Uttar Pradesh in India. These three geographies are settings where the Bill & Melinda Gates Foundation funds community based demand and supply side innovations to improve outcomes for mothers and newborns.[16] While these study areas are geographically diverse they each represent predominantly rural settings with high fertility and high maternal and newborn mortality (Table 2).

**Table 2:** Descriptive statistics for geographies included in this analysis.

Descriptive statistics	Gombe State, North East Nigeria	Ethiopia	Uttar Pradesh, India
Total population	2,768,452^1^	84,734,000^5^	199,812,341^6^
Rural population (%)	74%^11^	83%^5^	78%^6^
Religion			
Christian	17%^2^	63%^2^	0^2^
Muslim	83%^2^	37%^2^	10%^2^
Hindu	0^2^	0^2^	90%^2^
Adult literacy rate	34.5^3^	39^5^	67.6^6^
Total fertility rate	5.5^4^	4.8^5^	3.8^10^
Maternal mortality ratio	549/100,000 live births^7^	350/100,000 live births^8^	300/100,000 live births^9^
Neonatal mortality rate	37/1000^4^	31/1000^5^	50/1000^9^
Under 5 mortality rate	128/1000^4^	77/1000^5^	92/1000^9^

^1^ Nigerian Bureau of Statistics, Nigeria: Social Statistics of Nigeria, 2012

^2^ IDEAS baseline survey

^3^ The National Literacy Survey, June 2010, National Bureau of Statistics, Nigeria. www.nigerianstat.gov.ng

^4^ Nigerian Demographic and Health Survey preliminary report, 2013 http://dhsprogram.com/pubs/pdf/PR41/PR41.pdf

^5^ UNICEF (2011) Country factsheets. www.unicef.org/infobycountry/ethiopia_statistics.html

^6^ Indian population Census 2011, www.census2011.co.in/census/state/uttarpradesh.html

^7^ http://www.unicef.org/nigeria/ng_publications_advocacybrochure.pdf

[for north east Nigeria as a whole]

^8^ United Nations Maternal Mortality Estimation Inter-agency group http://www.maternalmortalitydata.org/

^9^ Annual health survey bulletin 2011–12: Uttar Pradesh. http://www.censusindia.gov.in/vital_statistics/AHSBulletins/files2012/Uttar%20Pradesh_Bulletin%202011-12.pdf

^10^ Population Reference Bureau at http://www.un.org/esa/population/meetings/EGM-Fertility2009/Haub.pdf

^11^Annual Abstract of Statistics 2011, National Bureau of Statistics, Nigeria.

In each of these study settings, baseline surveys were carried out in 2012 as part of an ongoing study to investigate the extent to which innovations that were intended to enhance contacts between families and frontline workers (community based volunteers for maternal and newborn health, and health staff at primary level health facilities) lead to an increase in the coverage of life-saving interventions; follow-up data collection in the same settings is planned for 2015. The study combined a cluster based household survey with a survey of the frontline health workers and facilities (health posts and primary health facilities) assigned to provide routine maternal and newborn health services to those households before implementation of the innovations. These baseline data are presented here.

### Sample selection

#### Gombe State, NE Nigeria

**Fig 1:**
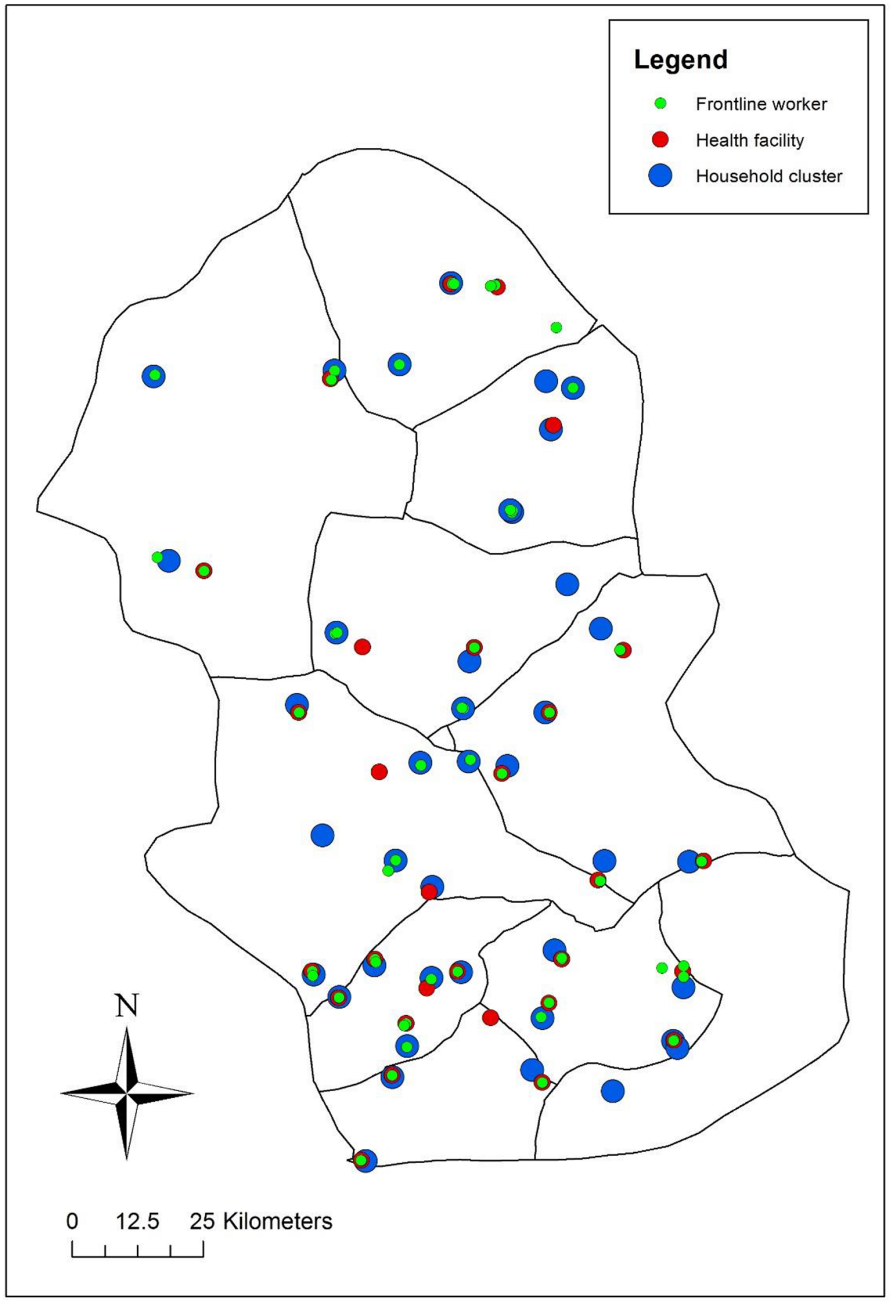
Map of study area in Gombe State, north east Nigeria showing location of household clusters, frontline workers, and primary health facilities surveyed.

Gombe State has 11 local government areas, and population statistics are available from the National Population Commission for enumeration areas throughout the State. The baseline survey included 40 clusters selected from 10 of the 11 local government areas in Gombe State (excluding Gombe Town). Clusters were defined as segmented enumeration areas. Cluster sampling was performed by listing all enumeration areas within the 10 local government areas, cumulating their population size, and systematically selecting 40 from the list with probability proportional to size. All households in selected enumeration areas were listed, and enumeration areas segmented into groups of 75 or fewer households: field teams randomly selected one segment from each enumeration area as the cluster to be surveyed. All households within the selected cluster were visited (Fig 1).

For each cluster, community level volunteers (for example, Traditional Birth Attendants or Federation of Muslim Women Association of Nigeria volunteers) were identified and listed and a simple random sample of up to 3 volunteers selected for interview about recent maternal and newborn health care they had provided. The primary health centre assigned to provide routine antenatal, intra-partum and post-natal care to the selected cluster was also surveyed, and the nurse who carried out the last delivery recorded in the maternity register interviewed (the frontline health worker and facility surveys).

#### Ethiopia

**Fig 2:**
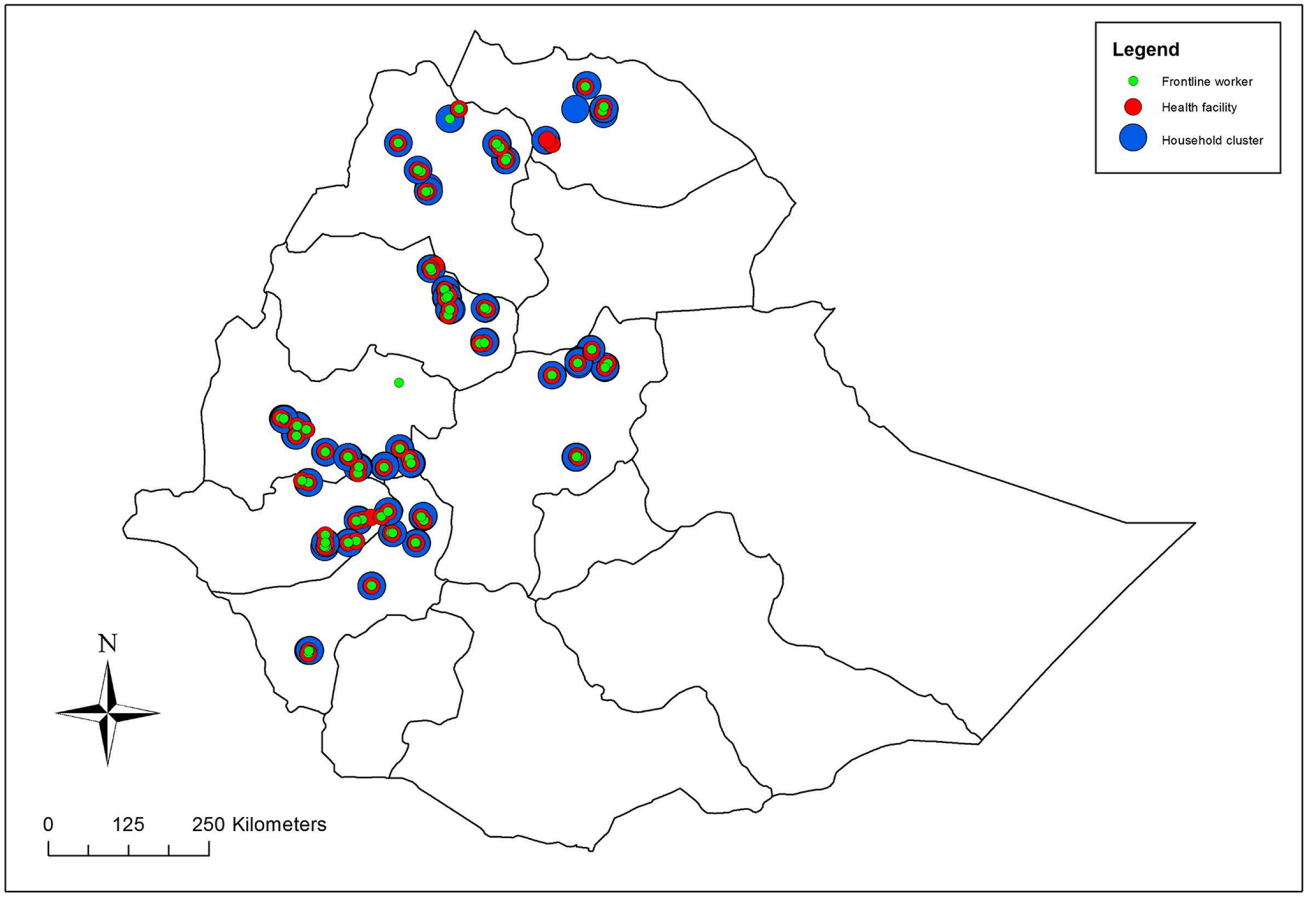
Map of study area in Ethiopia showing location of household clusters, frontline workers, and primary health facilities surveyed.

Ethiopia is organised by region, zone, *woreda* (district), *kebele* (similar to a ward; lowest level of census population data) and *gote* (proxy for village). The baseline survey included 80 clusters, a cluster being defined as a segmented gote. The 80 clusters were systematically sampled from 76 *woreda* across four regions of Ethiopia (Amhara, Oromia, SNNP and Tigray). Sampling was performed by listing all *woreda* geographically from north to south of the country, listing *kebeles* and their population size alphabetically within each *woreda*, and 80 *kebele* sampled with probability proportional to population size. *Gotes* within each of these 80 *kebele* were listed and one *gote* per *kebele* selected using simple random sampling. At each selected *gote*, all households were listed and *gotes* segmented into groups of 75 or fewer households: field teams randomly selected one segment from each *gote* as the cluster to be surveyed. All households within each selected cluster were visited (Fig 2).

For each sampled cluster, the community level volunteers were identified and listed and a simple random sample of up to 3 volunteers selected for interview about recent health care they had provided. The health post assigned to the village was surveyed, and the health extension worker on duty interviewed. The primary health centre assigned to provide routine antenatal, intra-partum and post-natal care to the selected cluster was also surveyed, and the nurse who attended the last delivery recorded in the maternity register interviewed (the frontline health worker and facility surveys).

#### State of Uttar Pradesh, India

**Fig 3:**
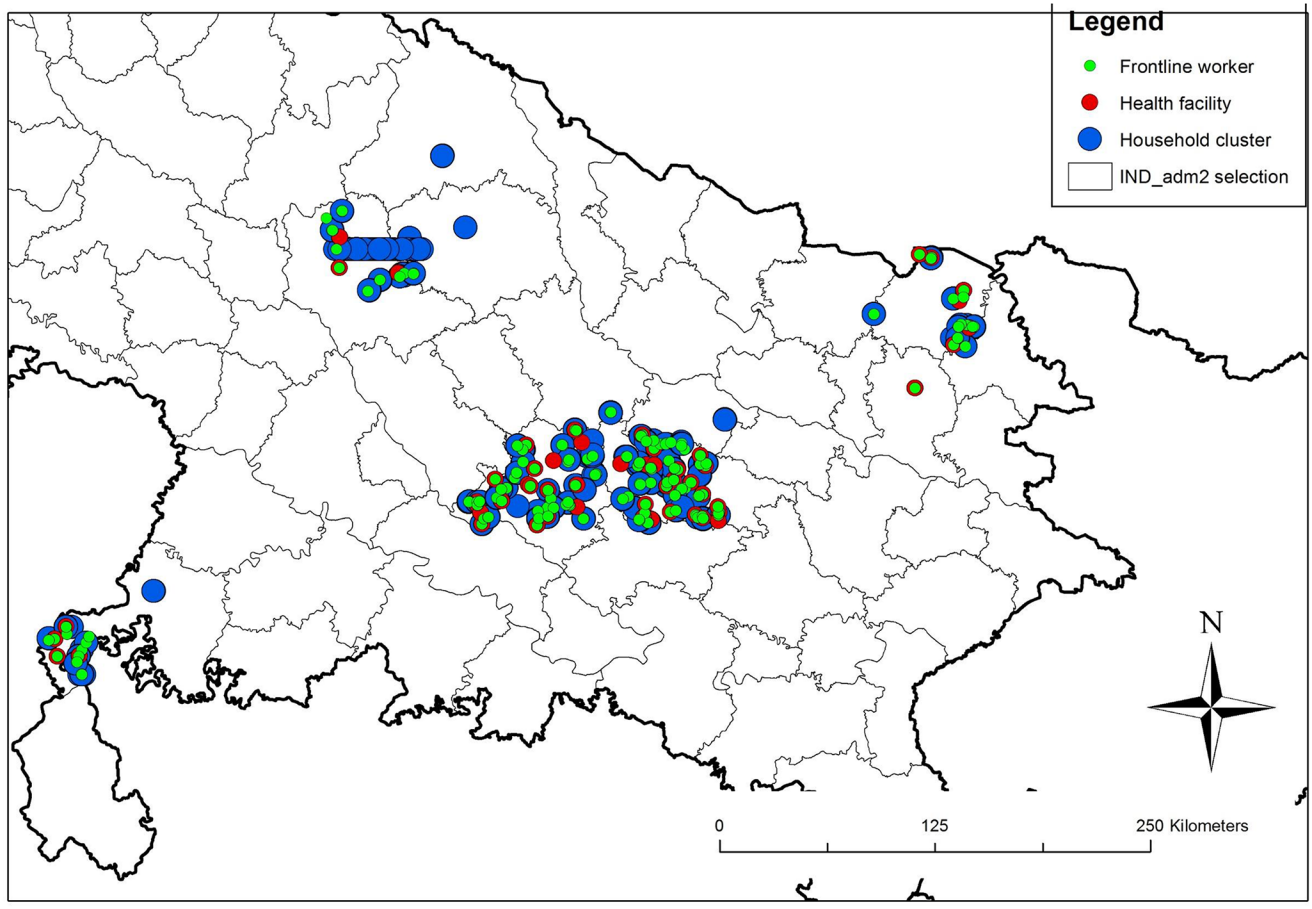
Map of study area in the State of Uttar Pradesh, India showing location of household clusters, frontline workers, and primary health facilities surveyed.

Uttar Pradesh is organised by district, block, and village. Population data is available at the village level from the 2001 Census. The baseline survey included 80 clusters, a cluster being defined as a segmented village. Sampling was performed by listing all villages from 51 blocks spread across six districts in Uttar Pradesh, cumulating their population size and systematically selecting 80 villages with probability proportional to size. All households in sampled villages were listed, and villages segmented into groups of 75 or fewer households: field teams randomly selected one segment from the selected village as the cluster to be surveyed. All households within the selected cluster were visited (Fig 3).

For each sampled cluster, the community level volunteers (Accredited Social Health Activists and Anganwadi workers) were identified and listed and a simple random sample of up to 3 volunteers selected for interview about recent maternal and newborn health care they had provided. The primary health centre or community health centre assigned to provide routine antenatal, intra-partum and post-natal care to the selected cluster was also surveyed, and the nurse who attended the last delivery recorded in the maternity register interviewed (the frontline health worker and facility surveys).

### Survey methods

Survey clusters were allocated to teams of five interviewers and one supervisor, and each cluster was scheduled to be completed within two working days. In Gombe State and Uttar Pradesh all data were collected using handheld personal digital devices (PDAs). In Ethiopia frontline health worker and health facility data were collected using PDAs and household data collected using paper based questionnaires, which were double entered and reconciled.

In all three countries the household questionnaire was modular and applied by interviewers who were allocated five to eight households per day depending on country and circumstance. At every household in the selected cluster the interviewer identified the household head or representative and, after obtaining informed written consent, recorded information about household socio-economic characteristics and completed a household roster of all usual residents. Every resident woman aged 13–49 years was then interviewed individually by the same interviewer and asked questions about her access to health care in the last year, and a detailed set of questions if she reported that she had had a live birth in the 12 months prior to survey.

The frontline health worker questionnaire included questions about training and supervision, routine activities carried out, availability of supplies, workload during the last month and a detailed set of questions about behaviours during the last birth they attended. The facility questionnaire included a check list of staff, equipment, drugs, and infrastructure items present on the day of survey, and data extraction from maternity registers to ascertain facility workload during the last six months.

All survey tools were pre-tested, and implemented in Hausa (Gombe State, Nigeria), Amharic (Amhara and SNNP regions, Ethiopia), Oromifa (Oromia region, Ethiopia) and Tigrinya (Tigray region, Ethiopia), and Hindi (State of Uttar Pradesh, India).

### Analysis

Data were analysed in STATA 12 (www.stata.com) using *svy* commands to adjust for the cluster sampling design. In each geography, the sample selection was expected to result in interviews with at least 350 women who had a live birth in the previous 12 months: this number was sufficient to estimate, with 90% power and 95% confidence, percentage points of coverage of all contact and content indicators across the continuum of care with approximately five percent precision, assuming a cluster design effect of 1.4.

Initially, contact and content indicators were calculated separately. Four contact coverage estimates were defined: (1) the percent of women with a live birth in the previous 12 months who had at least one antenatal care visit during that pregnancy; (2) the percent of women with a live birth in the previous 12 months who were attended at birth by a skilled birth attendant; (3) the percent of women with a live birth in the previous 12 months who had a post-partum check within 48 hours of birth; (4) the percent of newborns born alive in the previous 12 months whose mother reported that they had a post-natal check within 48 hours of birth. Amongst the same group of women with a live birth in the previous 12 months, the reported frequency with which each of the processes to define content of care (Table 1) occurred was calculated for all women, and then also calculated restricted to those women who had a contact.

Finally, point estimates for contacts were combined with those for processes to generate population level estimates of high quality contacts, being: (1) the percent of women who had at least one antenatal care visit and for whom all eight antenatal processes were met; (2) the percent of women who were attended at birth by a skilled birth attendant and received active management of third stage of labour (AMTSL); (3) the percent of women who had a post-partum check within 48 hours of birth and for whom all five post-partum processes were met; (4) the percent of newborns who had a post-natal check within 48 hours of birth and for whom all five post-natal processes were met.

These calculations were all carried out using household data with the exception of the intra-partum process AMTSL (which includes controlled cord traction, uterine massage, and administration of a prophylactic uterotonic). Women from the general population are often not able to report on the behaviours of birth attendants during delivery. To address this, efforts to link data sources have been proposed.[17, 18] In this analysis we linked skilled birth attendant responses about whether or not they had performed controlled cord traction, uterine massage and administered a prophylactic uterotonic at the last birth they attended (from the frontline health worker survey), to the household level coverage of delivery with a skilled birth attendant (household survey).

### Ethics

In Nigeria, national level approval was obtained from the National Health Research Ethics Committee, Federal Ministry of Health, Abuja, and in Gombe State from the State Ministry of Health in both Gombe and Abuja. In Ethiopia, national level support was obtained from the Ministry of Health in Ethiopia, and ethical approval from the Ministry of Science and Technology; at the Regional level, approval was granted by the Regional IRBs in Amhara, Oromia, SNNP, and Tigray. In Uttar Pradesh, approval was obtained from SPECT-ERB, an independent Ethical Review Board, and written permission from the National Rural Health Mission of Uttar Pradesh. Ethical approval was also obtained from LSHTM (reference 6088). All respondents provided informed, voluntary written consent to be interviewed. In addition, written carer consent was also obtained for the small number of household survey respondents under the age of 16 years.

## Results

The 2012 baseline surveys were carried out in June in Gombe State, May-July in Ethiopia, and November-December in Uttar Pradesh. In Gombe, Ethiopia and Uttar Pradesh respectively there was an average of 1.4, 1.0 and 1.6 resident women aged 13–49 years per household, 74%, 89%, and 93% of whom were interviewed, and 17%, 14%, and 7% of whom had a birth in the 12 months prior to the survey (Table 3). Non-response was due to women not being at home after the third visit to households; fewer than one percent of women refused to be interviewed. Sixty one frontline health workers were identified and interviewed in the 40 Gombe clusters (20 of whom were skilled birth attendants and contributed data to the intra-partum calculations); 316 in the 80 Ethiopian clusters (76 of whom were skilled birth attendants), and 217 in the 80 Uttar Pradesh clusters (55 of whom were skilled birth attendants).

**Table 3:** Study population in the three geographies and coverage of contacts between women and health care providers for maternal and newborn health care.

Study population:	Gombe State, North East Nigeria	Ethiopia	Uttar Pradesh,India
	N	N	N
Clusters	40	80	80
Households	1844	4285	5258
Resident women aged 13–49	2714	4398	8677
Interviewed women aged 13–49*	2021	3895	8092
Interviewed women with a live birth 12 months prior to survey	348	533	604
Coverage of contacts amongst women with a live birth 12 months prior to survey:	% (95% CI)	% (95% CI)	% (95% CI)
At least one antenatal care contact	61 (50–72)	56 (49–63)	74 (69–79)
At least 4 antenatal care contacts	40 (29–50)	23 (18–30)	28 (23–33)
Institutional delivery	30 (20–40)	14 (9–20)	76 (71–80)
Skilled attendant at birth	22 (14–29)	15 (11–22)	76 (71–80)
Post-partum check <48 hrs of birth	7 (4–9)	3 (2–6)	54 (48–59)
Post-natal check <48 hrs of birth	4 (2–8)	4 (2–6)	19 (15–23)

*Percent interviewed of all resident women: Gombe 74%, Ethiopia 89%, Uttar Pradesh 93%

Coverage of contacts in each geography are shown in Table 3. In Gombe, 61% of women had at least one antenatal care contact (95% confidence interval 50–72), 22% delivered with a skilled birth attendant (95% confidence interval 14–29), and fewer than 10% reported a health check on either themselves or their newborn within 48 hours of birth. A similar picture of contact coverage was observed amongst Ethiopian women. Contact coverage was much higher in Uttar Pradesh with 74% of women having at least one antenatal care contact (95% confidence interval 69–79), 76% having a skilled attendant at birth (95% confidence interval 71–80), 54% having a post-partum check within 48 hours of birth (95% confidence interval 48–59), and 19% of newborns having a post-natal check within 48 hours of birth (95% confidence interval 15–23) (Table 3).

### Content of antenatal care

In the household surveys, women who had any antenatal care were asked about the content of that care and results are shown in Table 4. In Gombe State, over three-quarters of women who had any antenatal care reported that they had their weight measured (87%, 95% CI 80–92), blood pressure measured (88%, 95% CI 81–93), and were counselled about breastfeeding (76%, 95% CI 67–83). However, only approximately one half had their height measured (53%, 95% CI 42–63) or their urine tested (54%, 95% CI 40–68). In Ethiopia, around two-thirds of women who had any antenatal care reported that they had their weight measured (72%, 95% CI 64–79), blood pressure measured (62%, 95% CI 56–68), and blood tested (68%, 95% CI 57–77). Just over one-third had their height measured (39%, 95% CI 32–47), urine tested (38%, 95% CI 31–46), or received counselling about danger signs (35%, 95% CI 28–43). In Uttar Pradesh, fewer than half of women reported any of processes being met as part of their antenatal care (Table 4).

**Table 4:** Reported content of antenatal care amongst all women with a live birth in the 12 months prior to survey, and amongst those women who had at least one antenatal contact with any provider during that pregnancy in Gombe State, Ethiopia, and Uttar Pradesh.

	Gombe State, NE Nigeria		Ethiopia		State of Uttar Pradesh, India	
	All women N = 348	Women with a contact N = 206	All women N = 533	Women with a contact N = 299	All women N = 604	Women with a contact N = 449
	% (95% CI)	% (95% CI)	% (95% CI)	% (95% CI)	% (95% CI)	% (95% CI)
*Reported content of antenatal care received at least once during pregnancy*						
Weight measured	56 (45–66)	87 (80–92)	45 (40–54)	72 (64–79)	32 (27–36)	43 (34–50)
Height measured	35 (26–44)	53 (42–63)	27 (22–32)	39 (32–47)	*Not recorded*	*Not recorded*
Blood pressure measured	57 (47–68)	88 (81–93)	41 (36–47)	62 (56–68)	34 (29–39)	41 (36–47)
Urine tested	36 (26–48)	54 (40–68)	24 (19–30)	38 (31–46)	33 (28–38)	39 (34–45)
Blood tested	45 (35–55)	68 (57–77)	47 (40–54)	69 (61–75)	24 (20–29)	28 (23–34)
Counselled about breastfeeding	49 (39–60)	76 (67–83)	34 (28–40)	46 (39–54)	28 (23–32)	30 (26–36)
Counselled about danger signs	41 (31–51)	64 (53–74)	24 (19–29)	35 (28–43)	28 (23–32)	31 (27–37)
Counselled about birth preparedness	46 (35–57)	73 (62–81)	36 (30–43)	52 (44–60)	29 (24–34)	34 (29–39)

**Fig 4:**
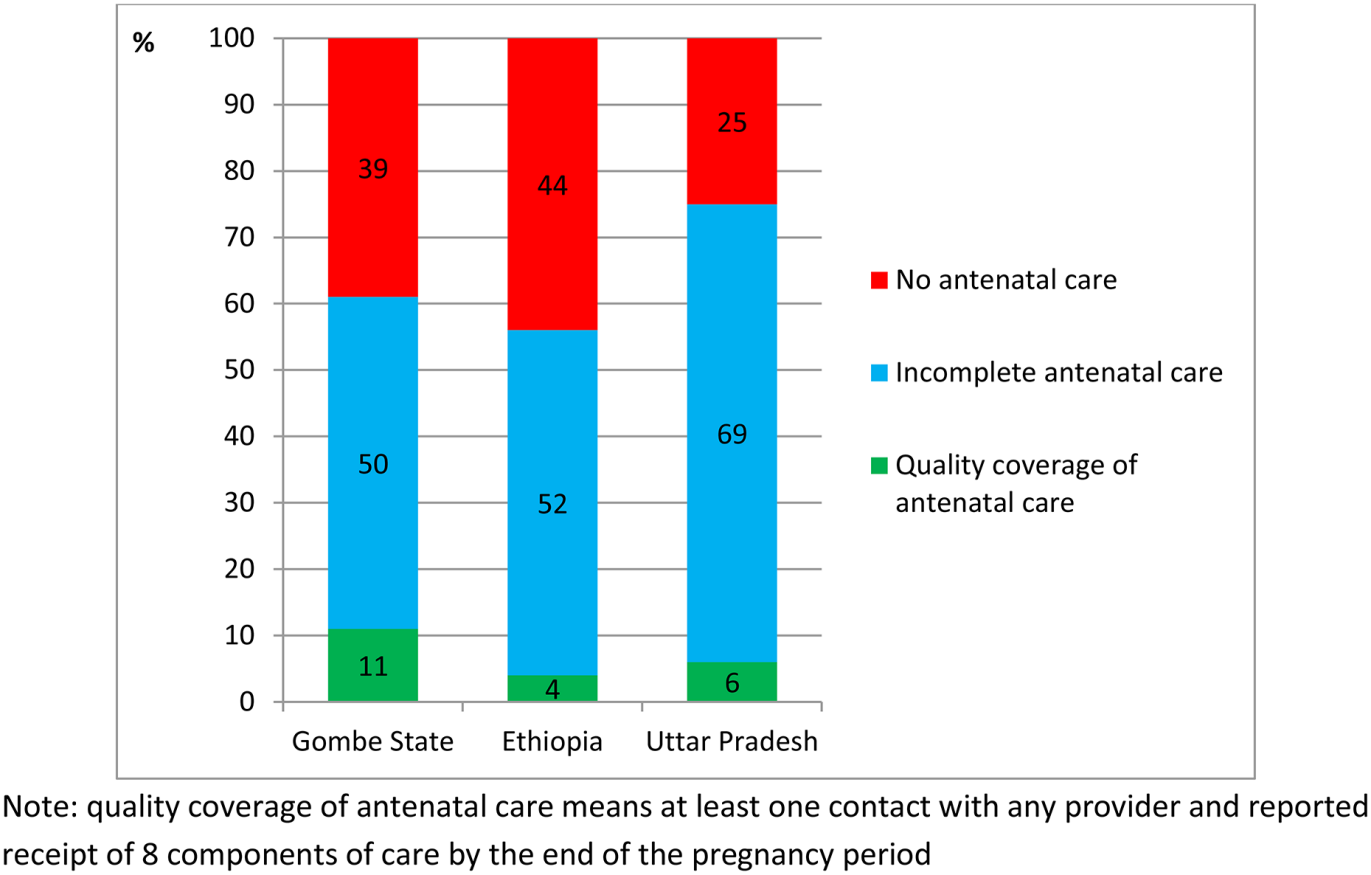
Coverage of quality antenatal care amongst women who had a live birth in the 12 months preceding survey in Gombe State, north east Nigeria, Ethiopia, and the State of Uttar Pradesh, India.

The reported coverage of antenatal care contact (Table 3) and content (Table 4) was combined to generate an estimate of the coverage of high quality antenatal care of 11% (95% CI 6–16) in Gombe State, 4% (95% CI 2–6) in Ethiopia, and 6% (95% CI 4–8) in Uttar Pradesh, Fig 4).

Because medical and counselling processes are qualitatively different, we also restricted analysis of quality antenatal care to include only the medical processes (thus excluding the three counselling processes about breastfeeding, danger signs, and birth preparedness). The resulting coverage estimates of high quality antenatal care were 17% in Gombe State, 9% (95% CI 6–13) in Ethiopia, and 13% (95% CI 9–16) in Uttar Pradesh.

### Content of intra-partum care

In the frontline health worker interviews, skilled birth attendants answered a detailed set of questions about their preparations and behaviours at the last birth attended. In Table 5 we present responses for the three AMTSL processes (controlled cord traction, uterine massage, and prophylactic administration of a uterotonic). In Gombe State, 60% (95% CI 40–77) reported that they administered a prophylactic uterotonic, 50% (95% CI 30–70) reported performing both controlled cord traction and uterine massage, resulting in an estimate of 35% (95% CI 19–56) of skilled birth attendants performing AMTSL at the last birth attended. In Ethiopia, 45% (95% 34–56) reported administration of a prophylactic uterotonic, 37% (95% CI 27–48) controlled cord traction, and 30% (95% CI 21–42) reported that they performed uterine massage, resulting in an estimate of 24% (95% CI 15–33) of skilled birth attendants performing AMTSL at the last birth attended. In Uttar Pradesh, 49% (95% CI 36–63) reported administration of a prophylactic uterotonic, 24% (95% CI 14–38) reported that they performed controlled cord traction, and 33% performed uterine massage (95% CI 22–46) resulting in an estimate of just five percent (95% CI 2–16) of skilled birth attendants performing AMTSL at the last birth attended (Table 5).

**Table 5:** Reported actions taken by skilled birth attendants at the last birth attended in Gombe State, Ethiopia, and Uttar Pradesh: Active Management of Third Stage Labour.

	Gombe State, NE Nigeria N = 20	Ethiopia N = 76	Uttar Pradesh, India N = 55
	% (95% CI)	% (95% CI)	% (95% CI)
Estimated AMTSL behaviour at last birth	35 (19–56)	24 (15–33)	5 (2–16)
Components of AMTSL:			
Administration of prophylactic uterotonic	60 (40–77)	45 (34–56)	49 (36–63)
Controlled cord traction	50 (30–70)	37 (27–48)	24 (14–38)
Uterine Massage	50 (30–70)	30 (21–42)	33 (22–46)

**Fig 5:**
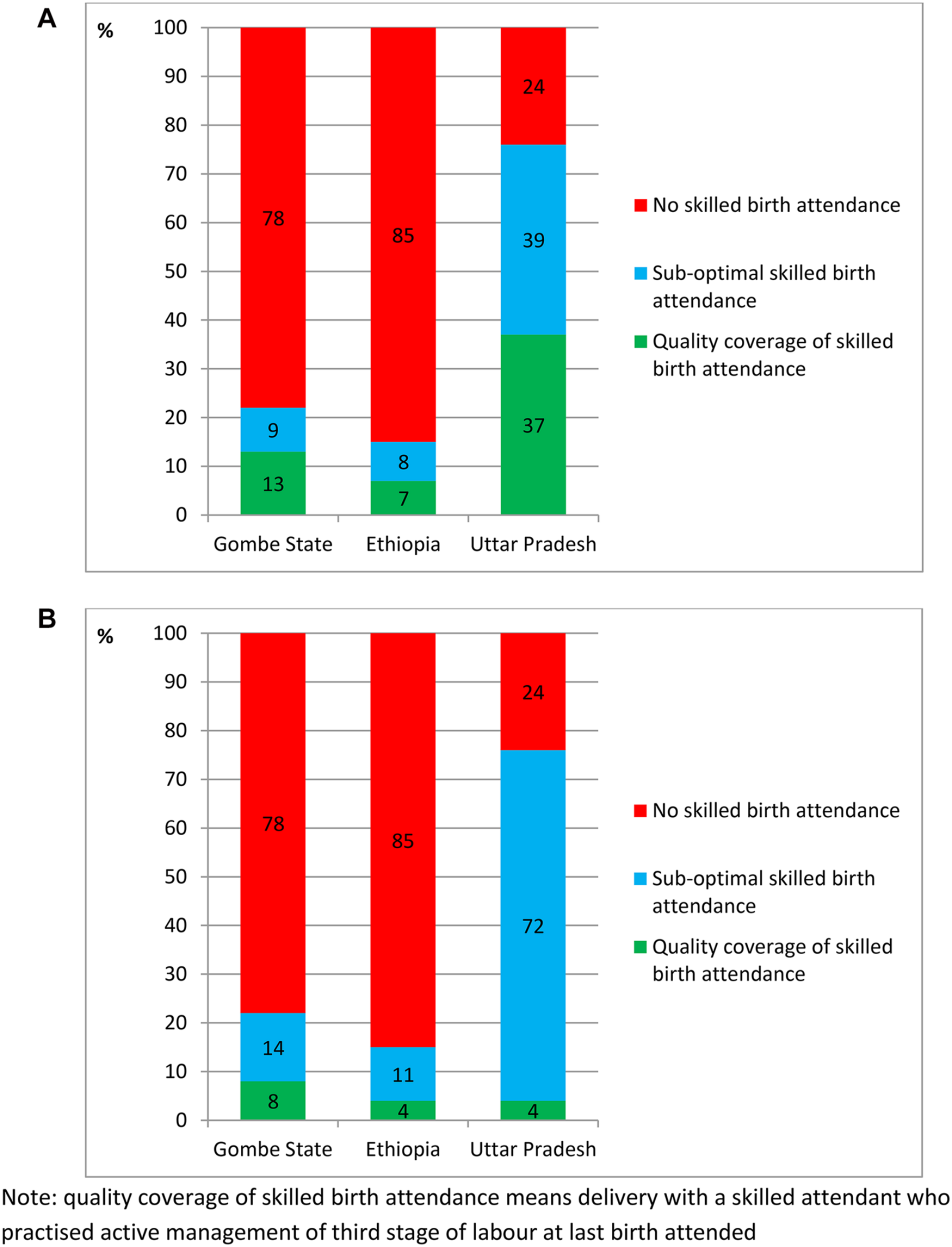
a. Coverage of quality skilled attendance at birth amongst women who had a live birth in the 12 months preceding survey in Gombe State, north east Nigeria, Ethiopia, and the State of Uttar Pradesh, India: defined as having a skilled attendant at birth and administration of prophylactic uterotonics. b. Coverage of quality skilled attendance at birth amongst women who had a live birth in the 12 months preceding survey in Gombe State, north east Nigeria, Ethiopia, and the State of Uttar Pradesh, India: defined as having a skilled attendant at birth and active management of third stage of labour.

These reported behaviours by skilled birth attendants (Table 5) were linked to coverage of delivery with a skilled birth attendant from the household survey (Table 3). The resulting coverage estimates of high quality intra-partum care (defined as skilled birth attendance with administration of prophylactic uterotonics to prevent post-partum haemorrhage) were 13% (95% CI 10–17) in Gombe, 7% (95% CI 5–9) in Ethiopia, and 37% (95% CI 34–42) in Uttar Pradesh, Fig 5a). The coverage estimates of high quality intra-partum care that included skilled birth attendance and all three components of AMTSL were 8% (95% CI 5–11) in Gombe State, 4% (95% CI 2–6) in Ethiopia, and 4% (95% CI 2–6) in Uttar Pradesh, Fig 5b).

### Content of post-partum care within 48 hours of birth

In the household surveys, women who reported post-partum care within 48 hours of birth were asked about the content of that care and results are shown in Table 6. In Gombe State, the most frequently reported process was nutrition counselling (reported by 35%, (95% CI 18–56) of women who had post-partum care), but all other processes were reported by fewer than 20% of women receiving care. In Ethiopia, the least frequently reported process was examination of breasts (39%, 95% CI 19–63%) and the most frequent process was checks on bleeding (56%, 95% CI 38–72). In Uttar Pradesh, just under half of women who had received post-partum care reported that their breasts had been checked (46%, 95% CI 39–53), one third reported receiving counselling about nutrition (38%, 95% CI 31–45) and danger signs (35%, 95% CI 29–41), and approximately one quarter reported receiving counselling about family planning (27%, 95% CI 22–33) and that their bleeding was checked (24%, 95% CI 18–30, Table 6).

**Table 6:** Reported content of post-partum care amongst all women with a live birth in the 12 months prior to survey, and amongst those women who had at least one post-partum contact within 48 hours of birth in Gombe State, Ethiopia, and Uttar Pradesh,

	Gombe State, NE Nigeria		Ethiopia		State of Uttar Pradesh, India	
	All women N = 348	Women with a contact N = 23	All women N = 533	Women with a contact N = 18	All women N = 604	Women with a contact N = 324
	% (95% CI)	% (95% CI)	% (95% CI)	% (95% CI)	% (95% CI)	% (95% CI)
*Reported content of post-partum care received at least once within 48 hours of birth*						
Breasts checked	3 (1–5)	17 (6–41)	4 (2–6)	39 (19–63)	28 (24–33)	46 (39–53)
Bleeding checked	1 (0–3)	17 (7–37)	3 (2–5)	56 (38–72)	15 (11–19)	24 (18–30)
Counselled about danger signs	0	0	2 (1–4)	39 (16–68)	21 (17–26)	35 (29–41)
Counselled about nutrition	5 (3–7)	35 (18–56)	4 (2–6)	44 (22–70)	21 (18–26)	38 (31–45)
Counselled about family planning	3 (1–5)	9 (2–30)	4 (2–6)	50 (27–73)	17 (13–20)	27 (22–33)

**Fig 6:**
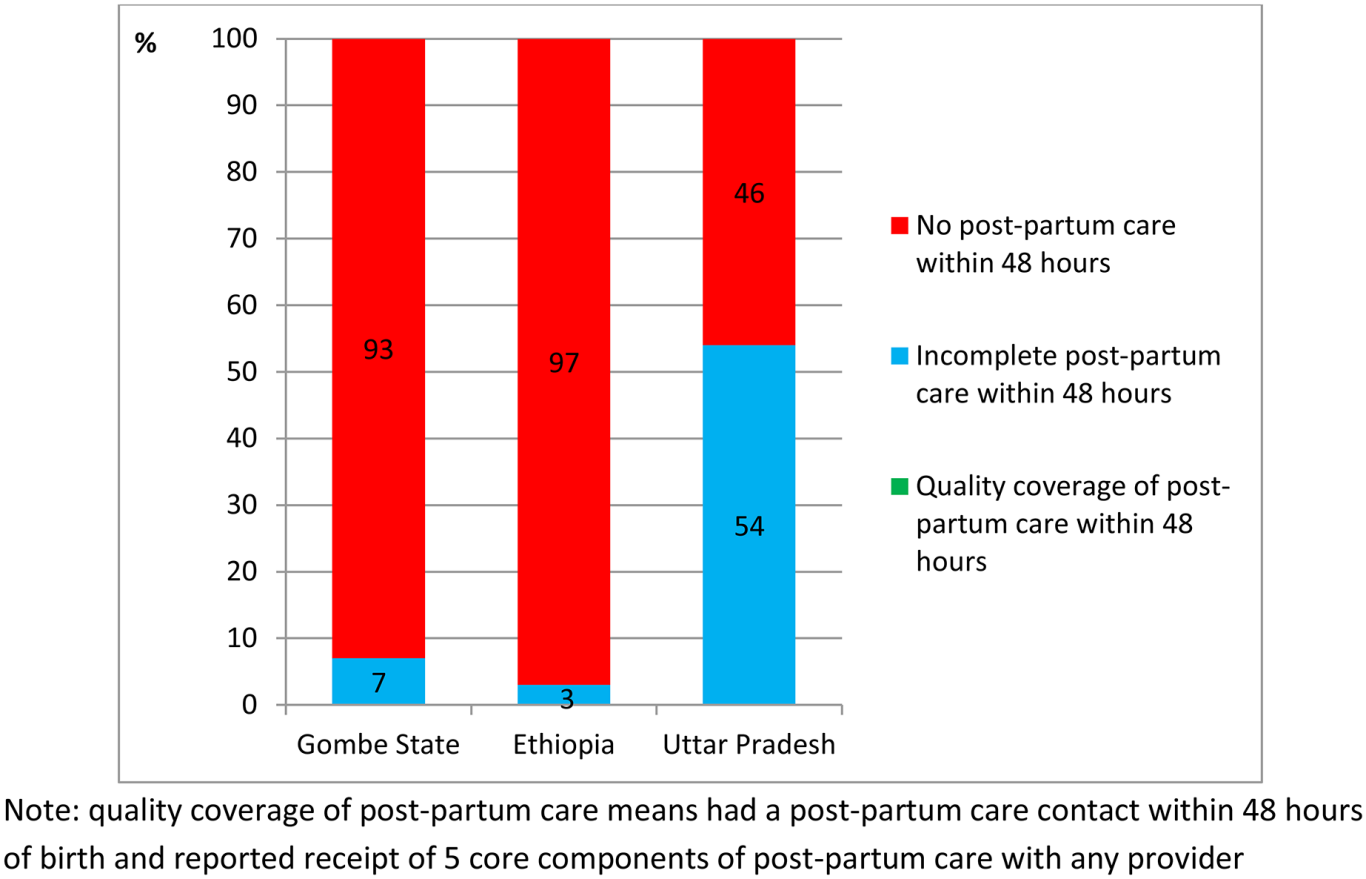
Coverage of quality post-partum care amongst women who had a live birth in the 12 months preceding survey in Gombe State, north east Nigeria, Ethiopia, and the State of Uttar Pradesh, India.

The reported coverage of post-partum care contact (Table 3) and content (Table 6) was combined to generate a coverage estimate for quality post-partum care of zero in all three countries (Fig 6).

### Content of post-natal care within 48 hours of birth

In the household surveys, women who reported that their newborn had a post-natal check within 48 hours of birth were asked about the content of that care and results are shown in Table 7. In Gombe State, the most frequently reported processes were newborns having their cord checked, and body examined for danger signs (29%, 95% CI 11–55), but no mothers reported receiving counselling about thermal care. In Ethiopia, three quarters of mothers reported that their newborn’s cord was checked (74%, 95% CI 47–90), two-thirds were counselled about breastfeeding (68%, 95% CI 42–87), the remaining process criteria being reported by one-third or fewer mothers. In Uttar Pradesh, approximately one half of mothers reported receiving counselling about breastfeeding (55%, 95% CI 46–64), and that their newborn’s cord was checked (54%, 95% CI 43–65) and 41% (95% CI 30–53) reported that their newborn was weighed. Thermal care counselling was reported by only 7% (95% CI 4–13) of mothers (Table 7).

**Table 7:** Reported content of post-natal care reported by all women with a live birth in the 12 months prior to survey, and amongst those women who reported that their newborn had at least one post-natal contact within 48 hours of birth in Gombe State, Ethiopia, and Uttar Pradesh.

	Gombe State, NE Nigeria		Ethiopia		State of Uttar Pradesh, India	
	All newborns N = 348	Newborns with a contact N = 14	All newborns N = 533	Newborns with a contact N = 19	All newborns N = 604	Newborns with a contact N = 114
	% (95% CI)	% (95% CI)	% (95% CI)	% (95% CI)	% (95% CI)	% (95% CI)
*Reported content of post-natal care received at least once within 48 hours of birth*						
Weight checked	2 (1–6)	14 (3–52)	2 (1–4)	32 (13–58)	8 (6–11)	41 (30–53)
Cord checked	3 (1–5)	29 (11–55)	4 (3–7)	74 (47–90)	11 (8–15)	54 (43–65)
Body examined for danger signs	3 (1–5)	29 (11–55)	2 (1–4)	26 (12–49)	2 (1–4)	12 (8–19)
Caregiver counselled about thermal care	1 (0–3)	0	2 (1–5)	37 (16–64)	1 (0–3)	7 (4–13)
Caregiver counselled about breast feeding	3 (1–5)	21 (6–53)	5 (3–8)	68 (42–87)	11 (9–15)	55 (46–64)

**Fig 7:**
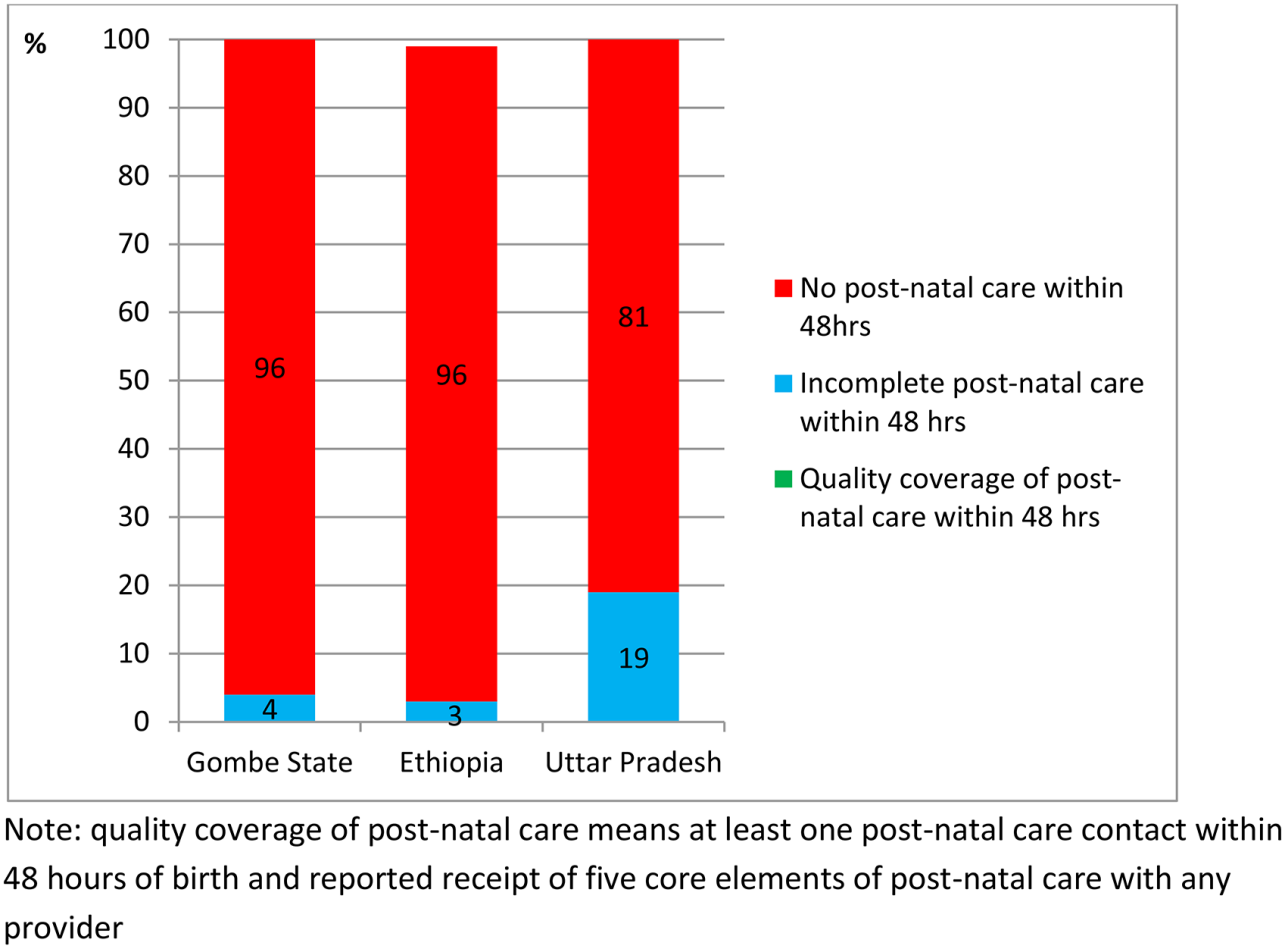
Coverage of quality post-natal care amongst infants born in the 12 months preceding survey in Gombe State, north east Nigeria, Ethiopia, and the State of Uttar Pradesh, India.

The reported coverage of post-natal care contact (Table 3) and content (Table 7) was combined to generate a coverage estimate for quality post-natal care of zero in all three countries (Fig 7).

## Discussion

Applying a measurement method that linked the content of care (defined by the routine processes recommended to take place at a given contact) to the frequency of those contacts revealed important areas of missed opportunity for optimising the health care received by women and their newborns. The three study sites to which this method was applied were very diverse, yet consistent patterns emerged. The coverage of high quality contacts for mothers and newborns in Gombe State in Nigeria, Ethiopia, and the State of Uttar Pradesh in India was considerably lower than the crude contact coverage estimates. Quality antenatal care coverage was reduced from over half of all women having contact to 11% or fewer women having contact with all the recommended routine content. Despite large variation in coverage of delivery with a skilled attendant between geographies, fewer than ten percent of women were estimated to have skilled birth attendance with recommended content (AMTSL). No women or newborns had a post-birth check up with complete recommended content. These findings are important for the development of strategies to optimise the life-saving potential of health care in the context of a world that aims to achieve universal health care.

Conceptually, our approach has been to separate three different stages in health care: (1) contacts (for example an antenatal care visit), (2) content of contacts (the recommended processes at each stage, for example measuring blood pressure, or testing for presence of protein in urine), then (3) delivery of life-saving interventions arising as a result of the contact and content (prescription of a hypertensive drug if indicated to manage preeclampsia). Our goal has been to estimate the population level coverage of the first two of these stages as an indicator of quality contacts; this approach is similar to that described as effective coverage, and the importance of building in measures of quality for maternal and newborn health has been discussed previously.[19–21] Some content processes were reported to occur more frequently than others and there were major variations across the three geographies. For example, conducting blood tests, and measuring blood pressure during antenatal care were much less common in Uttar Pradesh than the other two geographies. In interpreting these findings, it is useful to apply a quality improvement lens. Consideration of which processes could easily be changed, which are most important to change, and which actions would be required to change them in a given context, could help programmers to target individual actions that collectively result in large health gains.

The approach presented was implemented using data from a modular cluster-based household survey. Current large scale data collection approaches for maternal and newborn health tend to measure contacts, coverage of some life-saving interventions, and readiness to provide care at the health facility level, but until recently have put less emphasis on content of care received. While recognising the importance of understanding health system readiness to provide care, these approaches do not capture the necessary step of health worker behaviour.[22] For example, a nurse working in a health facility with access to a blood pressure cuff may not measure blood pressure during an antenatal consultation for reasons beyond availability of the equipment. Of the processes listed in Table 1, Demographic and Health Surveys currently ask women who had a recent pregnancy whether anyone checked her blood pressure, tested her urine or blood, or counselled her about danger signs. These surveys also ask whether the newborn was weighed at birth, and whether the caregiver was counselled about breastfeeding. With the possible exception of skilled birth attendance, extending analysis of household survey data to routinely estimate the percent of women and newborns who had contacts with recommended content could systematically reveal important variations in coverage of quality contacts both within and between countries.[23, 24]. Analysis of household survey data in this way could have particular relevance for answering questions about population level equity, a topic that routinely collected facility-based data cannot yet address.

These data have some limitations. First, the populations sampled were representative of a programme of work in each geography at baseline,[16] but were not designed to be representative at the country level. However our findings on the frequency of contacts from antenatal to post-natal care are consistent with most recent sub-national figures, but estimates for content of care are not routinely reported and are not easy to compare currently.[25–27] Second, we calculated content indicators at the population level, but also amongst women and newborns who accessed care in order to understand more about the quality of care they received. In 2012, the coverage of post-partum and post-natal contacts remained very low meaning that our sample size for examining the content of this type of care was small. Linked to this, no disaggregation of results by characteristic of respondent was possible. Nonetheless, given that none of the respondents reported receiving all processes for post-partum and post-natal care we consider these results worthy of highlight. Third, the quality of intra-partum care measures linked skilled birth attendant responses about their own behaviour at the last birth they attended with population level coverage of skilled birth attendance. Clearly data representing the behaviours of birth attendants for individual women would be preferable. Despite this limitation, we had justifications from the user and provider perspectives for using this approach. Although women have been demonstrated to reliably report on use of prophylactic uterotonics in health facilities,[28] to our knowledge that method has not been validated for population-based surveys; we felt it was very unlikely that women in the community could report on AMTSL behaviours. Birth attendants may be biased towards providing positive responses about their own behaviours, meaning that the method provides a ‘most optimistic’ estimate of coverage that can be applied in a standardised way. As we observe, results arising from this ‘most optimistic’ reporting fall far below the desired standard. Fourth, adjusting the coverage of contacts by coverage of recommended content of care is only one dimension of quality, as argued in the introduction, and more could be done to refine measures of high quality contacts (accepting this would likely reduce coverage estimates even further). Finally, all surveys of this type are susceptible to recall or responder bias,[29] and problems with missing data. In these surveys we interviewed women with a live birth in the 12 months prior to survey, a shorter recall than most national modular survey approaches,[23, 30] but the potential for women to mis-remember—or entirely forget events cannot be excluded. Use of PDAs for data capture can considerably facilitate the internal consistency and data quality[31] and missing data was not an important feature of these data.

In conclusion, measuring quality of care for mothers and newborns is complex but it was feasible to define and collect information about routine health care processes that represent the content of care, and to calculate the coverage of high quality contacts taking place in three diverse high mortality settings. In the context of increased demand for contacts with health care providers, unpacking and measuring supply side actions that maximise the potential for health gain is a priority.
